# 121 A Comparison of Large Language Models on First Aid Treatment of Minor Burns

**DOI:** 10.1093/jbcr/iraf019.121

**Published:** 2025-04-01

**Authors:** Audrey Stevens, Charles McCuskey, Arushii Nadar, Kareem Abdelfattah, Nicole Nnadi

**Affiliations:** Parkland Burn Center; University of Texas Southwestern Medical Center; University of Texas Southwestern Medical Center; Parkland Burn Center; University of Texas Southwestern Medical Center

## Abstract

**Introduction:**

Large language models (LLMs) are artificial intelligence tools that are trained to summarize, translate text, and respond to user questions that have grown in popularity since their release. While they have great potential to improve access to medical knowledge, there is also potential to spread misinformation due to lack of adequate accountability and review. Therefore, we sought to investigate the ability of LLMs to accurately and thoroughly answer basic burn wound care questions.

**Methods:**

We created a standardized list of prompts covering basic at-home burn first aid questions. This list encompassed what steps to take at home after a burn, what to avoid, and how to assess severity of burn and determine when one should seek the care of a doctor. These questions were then input into six different popular LLMs: ChatGPT, Claude, Perplexity, Copilot, Jasper, and Gemini. The responses were then collected and compiled by our team for review. Each set of responses was directly compared to the American Burn Association’s Recommendations for Initial First Aid Treatment of Minor Burns for accuracy (not containing any contradictory information to published guidelines) and thoroughness (containing all aspects of published guidelines).

**Results:**

While there were variations in the length and style of answers amongst the LLMs, all models responded with accurate description of burn depth and advice that aligned with the tenets of first aid treatment for minor burns including cooling the burn with cool but not cold water and avoiding ice, covering the burn with sterile bandage, and avoiding butter and home remedies. Only one LLM (ChatGPT) did not specify removing jewelry, rings, and clothing. All LLMs also gave accurate recommendations regarding when to seek medical attention, with only one (Gemini) not including burns over major joints. All LLMs recommended seeking advice from a healthcare provider with each prompt. References were given in only two LLMs (Copilot and Perplexity).

**Conclusions:**

The future of LLMs continues to develop but their growing use by both patients and medical providers is evident. Of the included LLMs, all provided data consistent with first aid guidelines for care of minor wounds without major inconsistencies or misinformation. While to be used with caution, the potential for LLMs to facilitate access to medical knowledge is great.

**Applicability of Research to Practice:**

The integration of LLMs into medical practice is inevitable. We must continue to investigate the applicability and accuracy as both providers and patients increasingly utilize them.

**Funding for the Study:**

N/A

